# Factors Affecting Job Satisfaction Among Diagnostic Radiography Technologists in Kuwait: A Mixed‐Methods Research Study

**DOI:** 10.1155/rrp/8811382

**Published:** 2026-05-18

**Authors:** Ebtehal M. Al-Qattan, Hamad Al-Hamad, Asseel Khalaf, Raed Saeed, Nabeel Akhtar

**Affiliations:** ^1^ Department of Radiologic Sciences, Faculty of Allied Health Sciences, Kuwait University, Kuwait City, Kuwait, kuniv.edu; ^2^ Department of Occupational Therapy, Faculty of Allied Health Sciences, Kuwait University, Kuwait City, Kuwait, kuniv.edu; ^3^ Department of Health Informatics and Information Management, Faculty of Allied Health Sciences, Kuwait University, Kuwait City, Kuwait, kuniv.edu

**Keywords:** depersonalization, emotional exhaustion, job satisfaction, personal accomplishment, radiography, technologists, turnover intention

## Abstract

**Background:**

Job satisfaction plays an important role in the retention of employees. The main factors that affect job satisfaction include nine components, which are payment, promotions, supervision, fringe benefits, contingent rewards, operating conditions, co‐workers, nature of work, and communication.

**Objective:**

This study aimed to determine the factors that affect the job satisfaction among diagnostic radiography technologists (DRTs) in Kuwait.

**Methods:**

The research utilized a mixed‐methods approach. Part I was a questionnaire study, which consisted of 32 questions, distributed electronically to the technologists in Kuwait’s hospitals. Part II involved 12 face‐to‐face semistructured interviews. The responses were collected and statistically processed using SPSS version 20.

**Results:**

The mean satisfaction level of DRTs was 136. Fifty‐one percent (51%) were satisfied, 37% were neutral, and the remaining 11.6% were dissatisfied. Nine domains were analyzed, and it was found that nationality, gender, and education significantly impacted satisfaction levels, whereas non‐Kuwaiti, males, and graduates with a B.Sc. showed higher levels of satisfaction (*p* < 0.05). The thematic analysis has determined three emerging themes, each with two subthemes, that provide a deeper understanding of the factors contributing to their work‐related well‐being. These themes are recognition and organizational support, professional growth and compensation, work environment, and educational impact.

**Conclusion:**

The general satisfaction among the DRTs in the Kuwaiti governmental hospitals was skewed to moderate to high satisfaction levels. The findings suggest that government policymakers should focus on improving promotion programs and career development for radiographers.

**Contribution:**

This paper provides insights into radiographers’ job satisfaction, guiding workforce policy and professional development initiatives.

## 1. Background

Job satisfaction is the extent to which individuals are content with their jobs, reflecting whether they like or dislike their work and how positively they evaluate their overall work experience [1]. It is widely recognized as a multidimensional construct that encompasses affective, cognitive, and contextual evaluations of work, and it has important implications for employee well‐being, motivation, organizational commitment, and performance [1–3]. In healthcare settings, job satisfaction is particularly important because it influences not only staff morale and retention but also service quality, teamwork, patient safety, and continuity of care [2–8]. Job satisfaction has multifaceted factors, which are interrelated and can be internal or external to each other. The internal factors may include performance and autonomy in the workplace. The external factors include factors such as pay, promotion, opportunities, and institutional communication as well as leadership style, recognition, workload, and organizational support [4, 5, 9–12].

Contemporary literature suggests that job satisfaction among healthcare professionals should not be understood as a single outcome driven only by salary or promotion. Rather, it emerges from the interaction between work demands and organizational resources, including supervision, communication, recognition, staffing, career progression, and professional identity [2–5, 9, 10, 12, 13]. Employees with high levels of job satisfaction tend to exhibit positive attitudes toward their jobs, resulting in reduced withdrawal behaviors. Conversely, low job satisfaction negatively affects workers and their attitudes toward both their jobs and their workplace [7, 8]. Moreover, dissatisfaction has been linked to increased levels of stress, exhaustion, and depression in the workplace [6]. Additionally, studies have found that job satisfaction is associated with burnout, stress, and healthcare workers’ intention to leave their jobs [2, 3, 7–10, 14]. In contrast, higher levels of job satisfaction in the healthcare sector are correlated with improved patient care and increased patient satisfaction [7].

In healthcare, occupational turnover is a critical issue, as it leads to the loss of highly trained professionals and imposes additional costs on institutions for hiring and training new staff. Studies have reported that low job satisfaction is one of the major causes of occupational turnover in the healthcare sector, resulting in the loss of experienced workers [7–10]. According to the literature, turnover and/or resignation are driven by two main factors: push and pull factors. Pull factors are positive influences, while push factors are negative conditions that can lower job satisfaction. Typically, push factors are internal to the organization and include elements such as management practices and institutional policies [8, 10]. Moreover, dissatisfied healthcare workers who remain in their roles may negatively impact the workplace. This can lead to reduced performance, increased withdrawal behaviors, and obstacles to organizational development [7, 8]. Accordingly, job satisfaction is relevant not only because of its effect on individual well‐being and retention, but also because it is closely linked to healthcare productivity, organizational effectiveness, and the sustainability of the workforce [1, 7, 8].

As literature shows, job satisfaction plays a critical role in employee retention. Understanding the factors that influence job satisfaction is therefore essential for improving staff stability and reducing turnover. In radiography, several factors have been identified as influencing job satisfaction. These include employment conditions, remuneration, working conditions, professional support, recognition, educational opportunities, and opportunities for career development [4, 5, 9–12, 15, 16]. Recent radiography literature has further emphasized the importance of workload intensity, burnout, under‐appreciation, communication, leadership, and work environment in shaping job satisfaction and workforce sustainability [2–5, 9, 10, 12, 17–20]. Recognition, motivation, and career development also play significant roles. For example, offering nuclear medicine technologists a clear professional identity and involving them in decision‐making processes may boost confidence, motivation, and long‐term commitment to the profession [11, 17]. Conversely, low income, vague job descriptions, and lack of professional support have been reported as key causes of dissatisfaction [5, 11, 15]. Burnout is another major concern and is commonly driven by emotional exhaustion, depersonalization, and reduced personal accomplishment and commitment to the profession [2, 3, 14].

Several studies reveal varying levels of job satisfaction. For instance, a study conducted in Saudi Arabia reported moderate to high job satisfaction among radiographers, particularly in relation to coworker relationships and some organizational factors [4]. A study of magnetic resonance technologists highlighted a strong correlation between job satisfaction and effective leadership models [12]. More recent evidence has shown that high workload and under‐appreciation are major contributors to burnout and low job satisfaction among radiographers, while radiography workforce shortages and retention concerns continue to affect service sustainability internationally [3, 18, 20]. In contrast, several studies have shown widespread dissatisfaction among radiologic technologists and radiographers, particularly in relation to pay, promotion, role clarity, and workplace support [5, 13–16]. In Jordan, emotional exhaustion was identified as a major factor related to dissatisfaction and burnout [14], while in Finland, researchers recommended several strategies to improve job satisfaction, including clearer job descriptions, improved work environments, better information systems, and more effective scheduling practices [15]. The most recent study from Morocco also suggested that job satisfaction may vary across imaging specialties, indicating that local work environments, patient interactions, and career structures may influence radiographers’ experiences differently [17].

Limited research has been conducted on radiographers and the factors influencing job satisfaction in the Gulf region and, more specifically, in Kuwait [4, 5, 11]. Job dissatisfaction can lead to poor service delivery, decreased productivity, and a higher risk of medical errors [18]. This, in turn, contributes to work‐related burnout, which negatively affects both individuals and organizations. Job dissatisfaction not only compromises professional performance but also undermines the quality of patient care [14]. Burnout, often stemming from job dissatisfaction, is a critical concern in healthcare. Burnout is characterized by emotional exhaustion and can be exacerbated by factors such as pressure at work, large workloads, and long shifts [2, 3, 14]. Emerging research also shows that workforce sustainability in radiography is increasingly shaped by broader professional issues, including retention, role development, interprofessional collaboration, and future role expectations [9, 19, 20]. Therefore, this study aimed to measure job satisfaction among diagnostic radiography technologists (DRTs) in Kuwait, identify the factors contributing to their overall job satisfaction, and explore how job satisfaction can be improved within the Kuwaiti public healthcare system.

## 2. Methods

### 2.1. Study Design, Period

This study employed an analytical cross‐sectional design conducted between April 2023 and August 2023, using a convergent mixed‐methods approach. Both qualitative and quantitative data were collected concurrently and then analyzed separately before integration at the interpretation stage [21, 22]. A convergent design was considered appropriate because it allowed the researchers to examine the breadth of job satisfaction quantitatively while also exploring participants’ lived experiences and explanations qualitatively [21].

### 2.2. Inclusion and Exclusion Criteria

The target population for this study consisted of all DRTs currently employed in public healthcare facilities under the Ministry of Health (MOH) in Kuwait, regardless of their educational level, nationality, religion, or ethnic origin. Participants were eligible for inclusion if they were actively working in radiology departments within MOH general hospitals and were under the age of 60, which is the official retirement age in Kuwait. There were no restrictions based on years of professional experience.

Individuals were excluded from the study if they were not employed in MOH general hospitals—for example, those working in private healthcare facilities or specialized institutions. Additionally, any responses submitted without informed consent or with incomplete data were excluded from the final analysis.

### 2.3. Sample Size

The survey was distributed to 314 DRTs using a convenient sampling technique, but four questionnaires were excluded because they lacked consent. According to the latest census from the MOH in 2018, the population of DRTs in Kuwait is 1090. A sample size of 285 or more is considered representative, providing a 95% confidence level that the true value lies within ±5% of the measured population value.

### 2.4. Study Setting: Participants and Recruitment

This research was conducted in public hospitals across Kuwait, targeting DRTs employed under the MOH. The study included participants from all general governmental hospitals, encompassing a broad representation of technologists from diverse demographic and professional backgrounds. Participants were recruited electronically through professional networks and social media platforms, where the survey link was distributed directly to eligible technologists. A purposive sampling strategy was used for the qualitative phase, selecting participants from those who had already completed the questionnaire and expressed willingness to be interviewed.

### 2.5. Data Collection and Instrument (Data Collection Technique and Tools)

A convergent parallel mixed‐methods design was employed. In this design, quantitative and qualitative data were collected simultaneously, analyzed independently, and then integrated to provide a more comprehensive understanding of job satisfaction among DRTs. This approach is particularly useful for validating quantitative findings with qualitative insights or gaining deeper context around complex issues [21, 22].

Quantitative data were collected using a validated questionnaire originally developed by Paul E. Spector and further supported by subsequent validation work in healthcare settings [1, 23, 24]. The instrument consists of 32 items rated on a six‐point Likert scale, ranging from strongly disagree to strongly agree. It covers eight key domains: pay, promotion, supervision, fringe benefits, coworkers, nature of work, communication, and general satisfaction. Additional questions collected demographic information and education level for statistical analysis. The Job Satisfaction Survey has been widely used across health‐related professions and has demonstrated acceptable psychometric properties in previous studies [1, 23, 24]. The questionnaire was digitally administered using Google Forms and shared via social media applications and professional networks to reach technologists in all public hospitals across Kuwait. Responses were collected electronically, anonymized, and prepared for statistical analysis.

The qualitative phase consisted of semistructured one‐on‐one interviews with a purposive sample of 12 DRTs who had completed the survey. The interview guide was developed based on the study objectives and emerging quantitative patterns and included open‐ended questions focusing on recognition, communication, workload, compensation, autonomy, and career development. As suggested by Kallio et al., this approach allows for a flexible yet focused exploration of specific domains, ensuring both structure and openness [25]. Each interview was conducted in a private, quiet setting to ensure confidentiality and comfort. Interviews were audio‐recorded, transcribed verbatim, and analyzed using thematic analysis, allowing patterns and themes to emerge from the participants’ narratives [22]. The integration of qualitative and quantitative findings was guided by mixed‐methods principles that emphasize complementarity, convergence, and expansion of findings [21, 26]. (see​ Table 1).

**TABLE 1:** Themes and subthemes from qualitative analysis.

Theme	Subtheme	Description	Example quote
Theme 1: Recognition and Organizational Support	Subtheme 1.1: Recognition and Reward	Desire for recognition and reward is a crucial aspect of job satisfaction, especially among non‐Kuwaiti DRTs.	“When we receive a simple ‘thank you’ or are mentioned in departmental meetings, it makes a significant difference” (Participant 1)
	Subtheme 1.2: Communication and Support	Satisfaction is linked to transparent communication about roles and departmental decisions.	“Knowing what is happening within the department and that our feedback is considered makes us feel part of the team” (Participant 4)

Theme 2: Professional Growth and Compensation	Subtheme 2.1: Career Development Opportunities	Opportunities for professional growth are especially valued by those with 2–5 years of experience.	“Continuous learning and chances to advance are what keep me motivated” (Participant 4)
	Subtheme 2.2: Salary and Benefits	Salary and benefits are significant satisfaction factors; concerns about fair compensation.	“When I consider the hours, we put in and the complexity of our tasks, I don’t feel the salary fully compensates us” (Participant 7)

Theme 3: Work Environment and Educational Impact	Subtheme 3.1: Workload and Autonomy	Balance between workload and autonomy; dissatisfaction in the mid‐career stage without proportional autonomy.	“The workload is manageable, and I have the autonomy to make decisions about how I handle my tasks” (Participant 5)
	Subtheme 3.2: Educational Impact	Educational attainment tied to satisfaction; mismatch between qualifications and roles for higher degree holders.	“Having a master’s degree, I expected more advanced responsibilities. I’m looking for roles that challenge me” (Participant 2)

### 2.6. Data Analysis

Descriptive statistics were employed to analyze variables using Statistical Package for the Social Sciences software (SPSS V.20). Chi‐square tests were employed to compare responses to different items across demographic variables such as gender, nationality, and level of education. Thematic analysis was employed to identify, analyze, and report patterns within the qualitative data [22]. This analytic strategy is consistent with prior mixed‐methods studies examining job satisfaction and related workplace experiences among healthcare professionals [26, 27].

## 3. Results

A total of 310 DRTs participated in the study. The demographics and job‐related characteristics are shown in Table 2. About 45.5% of participants were males, and 54.5% were females. Over half of the study participants were non‐Kuwaitis (64.5%). The largest group of experience was working for more than 10 years (41%). Most participants were bachelor’s degree holders (91.3%) (see Table 2).

**TABLE 2:** Socio‐demographic characteristics of study participants.

Gender	*n* (%)
Male	141 (45.5)
Female	169 (54.5)

*Nationality*	
Kuwaiti	110 (35.5)
Non‐Kuwaiti	200 (64.5)

*Years of experience*	
< 2 Years	43 (13.9)
> 10 Years	127 (41)
2–5 Years	60 (19.4)
6–10 Years	80 (25.8)

*Education*	
BSc degree	283 (91.3)
MSc degree	23 (7.4)
PhD degree	4 (1.3)

Table 2 summarizes the distribution of participants by gender, nationality, years of experience, and educational qualifications.

Descriptive analysis was done to measure the mean score of salary, promotion, supervision, fringe benefit, coworker, tasks, communication, and general satisfaction among different nationalities. Table 3 summarizes the responses of DRTs to the job satisfaction main domains. The maximum percentage value is 65.2%, which represents the satisfaction of DRTs with the task domain. Moreover, 35.6% of DRTs are neutral about the promotion processes, while 34.4% of DRTs are not satisfied with their salary. However, 33% of the DRTs are satisfied with the salary. The minimum value is 6.3%, which represents the dissatisfaction toward the coworkers.

**TABLE 3:** Distribution of DRTs related to job satisfaction with main domains: this table presents the levels of job satisfaction (satisfied, neutral, not satisfied) reported by diagnostic radiographic technologists (DRTs) across various job domains, where *n* is the cumulative item level.

No.	Domains	Satisfied		Neutral		Not satisfied	
	*n* = responses	*n*	%	*n*	%	*n*	%
1	SALARY	409	33.0^∗∗^	404	32.6	427	34.4
2	SUPERVISION	807	65.1	296	23.9	137	11.0
3	FRINGE BENEFITS	434	35.0	437	35.2	369	29.8
4	COWORKERS	724	58.4	438	35.3	78	6.3^∗∗^
5	COMMUNICATION	666	53.7	421	34.0	153	12.3
6	PROMOTION	514	41.5	442	35.6^∗^	284	22.9
10	TASKS	808	65.2^∗^	338	27.3	94	7.6
29	GENERAL SATISFACTION	699	56.4	384	31.0	157	12.7

^∗^The highest percentage value within the domain.

^∗∗^The lowest percentage value within the domain.

**FIGURE 1:**
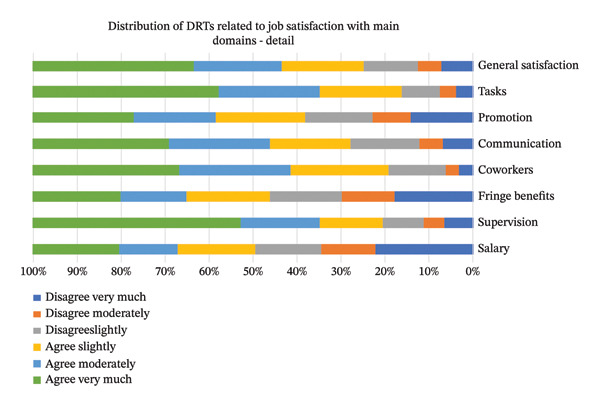
Detailed distribution of perceived job satisfaction across main domains. This bar chart illustrates the percentage of respondents indicating varying levels of agreement/disagreement (from “disagree very much” to “agree very much”) with statements related to job satisfaction across eight key domains: general satisfaction, tasks, promotion, communication, coworkers, fringe benefits, supervision, and salary. The breakdown provides a granular view of perceptions within each domain.

**FIGURE 2:**
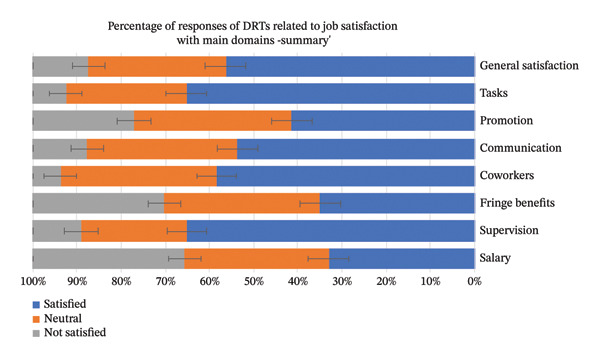
Summary of perceived job satisfaction across main domains. This bar chart presents a summarized view of job satisfaction, categorizing responses into “satisfied,” “neutral,” and “not satisfied” for each of the eight main domains (general satisfaction, tasks, promotion, communication, coworkers, fringe benefits, supervision, and salary).

Thirty‐two subdomains of job satisfaction were investigated. Figure 1 of the detailed distribution of the DRTs related to job satisfaction with the main domains shows that 50% of DRTs very much agreed with their supervisors, while 1.9% of the DRTs moderately disagreed, which has the lowest percentage toward the co‐worker’s subdomain. Additionally, 2.6% strongly disagreed with enjoying dealing with coworkers. Figure 2 summarizes the responses of DRTs related to job satisfaction with the main domains. The general satisfaction indicates 56.4% were satisfied, 31% were neutral, and 12.7% were not satisfied.

### 3.1. Qualitative Thematic Analysis

For the qualitative approach, 12 DRTs participated in the qualitative part of this study. Their demographic profiles are as follows: gender distribution: 4 males, 8 females. The age range is 25–60 years. The years of experience are 4–20 years. The workplace settings are governmental hospitals, including Al‐Adan Hospital, Al‐Razi Hospital, Mubarak Al‐Kabeer Hospital, and Al‐Sabah Hospital. While the quantitative data provided insight into the demographics and job‐related characteristics, the qualitative thematic analysis offered a deeper understanding of the personal experiences related to job satisfaction among DRTs. This analysis revealed three emerging themes, each with two subthemes, that significantly influence job satisfaction.

### 3.2. Theme 1: Recognition and Organizational Support

*Subtheme 1.1*: Recognition and Reward: Participants frequently mentioned the desire for recognition and reward as a crucial aspect of job satisfaction. Non‐Kuwaiti DRTs often discussed feeling valued when their work was acknowledged, which was reflected in their higher job satisfaction scores. One technologist stated, “When we receive a simple ‘thank you’ or are mentioned in departmental meetings, it makes a significant difference” (Participant 1).

*Subtheme 1.2*: Communication and Support: Effective communication and support from the organization emerged as a pivotal theme. Many reported satisfactions when there was transparent communication about job roles and decisions affecting their work. A participant shared, “Knowing what is happening within the department and that our feedback is considered makes us feel part of the team” (Participant 4).

### 3.3. Theme 2: Professional Growth and Compensation

*Subtheme 2.1*: Career Development Opportunities: Opportunities for professional growth were highlighted, particularly among those with 2–5 years of experience who exhibited the highest satisfaction levels. Those with longer tenure expressed a need for advanced career development to maintain job satisfaction. “Continuous learning and chances to advance are what keep me motivated,” noted a DRT with a decade of experience (Participant 4).

*Subtheme 2.2*: Salary and Benefits: Consistent with the quantitative findings, salary and benefits emerged as significant factors affecting job satisfaction. While some DRTs were satisfied with their salary, others highlighted discrepancies and sought better alignment with their responsibilities and market standards. “When I consider the hours, we put in and the complexity of our tasks, I don’t feel the salary fully compensates us. It’s not just about numbers; it’s about feeling valued for the work we do” (Participant 7).

### 3.4. Theme 3: Work Environment and Educational Impact

*Subtheme 3.1*: Workload and Autonomy: The balance between workload and autonomy was a recurrent topic. Participants with more than 10 years of experience felt they managed their workload effectively, which corresponded with their higher experience level. In contrast, those in the 6–10‐year bracket expressed dissatisfaction related to increased responsibilities without proportional autonomy or support. “The workload is manageable, and I have the autonomy to make decisions about how I handle my tasks. That autonomy is crucial for my job satisfaction” (Participant 5).

*Subtheme 3.2*: Educational Impact: Educational attainment was closely tied to job satisfaction, with those holding B.Sc. degrees expressing a sense of achievement and contentment with their roles. This was in stark contrast to the few master’s degree holders, who expressed dissatisfaction, which may be attributable to the underutilization of their qualifications or a mismatch in job expectations. “Having a master’s degree, I expected more advanced responsibilities. I’m looking for roles that challenge me and align better with my qualifications” (Participant 2) (see Table 4).

**TABLE 4:** Integrated quantitative and qualitative findings.

Themes and subthemes	Quantitative data	Qualitative data
Recognition and Organizational Support		Recognition is greatly valued; participants emphasized appreciation and acknowledgment from management.
Recognition and Reward	High satisfaction with supervisors (65.1% satisfied).	Transparent and regular communication was highly valued and significantly influenced feelings of inclusion.
Communication and Support	Moderate satisfaction with communication (53.7%).	
Professional Growth and Compensation		Opportunities for professional growth are highly desired; lack of advancement negatively impacts satisfaction.
Career Development Opportunities	Highest satisfaction among technologists with 2–5 years’ experience.	Salary dissatisfaction prominent, particularly regarding equity and alignment with responsibilities.
Salary and Benefits	34.4% expressed dissatisfaction regarding salary and benefits.	
Work Environment and Educational Impact		Autonomy is valued by experienced technologists, though workload pressures negatively impacted satisfaction for mid‐level staff.
Workload and Autonomy	High task satisfaction (65.2%), autonomy is positively viewed by seniors.	Higher‐degree holders (M.Sc., Ph.D.) expressed dissatisfaction due to perceived underutilization of their qualifications.
Educational Impact	Higher satisfaction among B.Sc. holders (*M* = 137.9).	

## 4. Discussion

This study addresses a significant gap in the literature, as there have been no previous mixed‐methods studies conducted specifically on job satisfaction among DRTs in Kuwait. By combining quantitative and qualitative findings, the present study extends current radiography and healthcare workforce literature by showing that job satisfaction among DRTs in Kuwait is shaped by multiple interacting domains rather than a single determinant [2–5, 9–12, 17–21, 26, 27]. A key strength of the study is the use of a comprehensive assessment approach to evaluate employee and workplace factors that are relevant to organizational performance, professional well‐being, and retention [1, 21, 23, 24]. This multifaceted approach suggests that job satisfaction arises from a combination of individual employee factors and broader workplace conditions.

The sociodemographic characteristics of the 310 DRTs who participated in the study showed that 45.5% of the participants were male and 54.5% were female. Regarding gender analysis, males had higher job satisfaction scores than females, and this difference was statistically significant. Although the reasons for this difference cannot be conclusively established from the present data, contemporary job satisfaction literature suggests that satisfaction patterns may reflect differences in role expectations, support, workload distribution, and access to career opportunities [6, 18, 28]. The highest level of satisfaction in males was in supervision and tasks, whereas females reported relatively lower scores in salary, fringe benefits, and promotion.

The nationality results showed a significant difference between Kuwaitis and non‐Kuwaitis, with non‐Kuwaitis reporting a higher level of job satisfaction. This finding may reflect contextual employment expectations, perceived job security, and differences in comparative benchmarks for compensation and workplace benefits. It also aligns with broader healthcare workforce literature showing that job satisfaction may vary across groups according to institutional support, perceived opportunity, and reward structures [7, 8, 18, 28]. However, these interpretations should be approached cautiously and examined further in future studies.

Most participants had more than 10 years of experience, although the highest overall satisfaction was found among those with 2–5 years of experience. While differences by years of experience were not statistically significant, the qualitative findings suggest that mid‐career technologists may experience a mismatch between increasing responsibility and insufficient recognition, support, or advancement opportunities. This interpretation is consistent with recent mixed‐methods and radiography literature emphasizing the importance of career development, recognition, and organizational support in sustaining job satisfaction over time [9, 10, 26, 27].

In terms of education, most participants held a bachelor’s degree, and B.Sc. holders had higher satisfaction levels than those with MSc or PhD qualifications. This pattern may indicate a mismatch between qualifications and role expectations, with more highly educated DRTs potentially perceiving underutilization of their skills or fewer opportunities for advanced professional roles. Similar concerns have been described in workforce and professional development literature, where alignment between qualifications, role scope, and advancement opportunities is closely related to job satisfaction [19, 20, 28, 29].

Overall, the present findings indicate that supervision, tasks, and coworker relationships were domains with relatively high satisfaction, whereas salary, fringe benefits, and promotion were domains with comparatively lower satisfaction. These results are broadly consistent with both earlier and contemporary radiography literature, which repeatedly identifies leadership, work environment, coworker support, recognition, pay, promotion, and burnout as central determinants of job satisfaction [2–5, 9–12, 17, 18]. Notably, the more recent evidence strengthens the interpretation of our findings by showing that workload and underappreciation remain especially important drivers of low satisfaction and burnout in radiography practice [3].

The qualitative findings both supported and enriched the quantitative results. While the survey demonstrated moderate overall satisfaction, interviews revealed deeper concerns related to recognition, communication, career growth, and workload. This added value is one of the main advantages of mixed‐methods research, as qualitative data help explain why apparently acceptable quantitative scores may coexist with strong dissatisfaction in specific domains or subgroups [21, 26, 30]. In the present study, thematic analysis identified three main themes: recognition and organizational support, professional growth and compensation, and work environment and educational impact. These themes align closely with current scholarship in healthcare and radiography, which views job satisfaction as influenced by both measurable conditions and less tangible experiences such as feeling valued, being heard, and having a clear professional future [2–5, 9, 17–20, 27, 29].

The theme of recognition and organizational support was particularly prominent. Participants emphasized appreciation, transparent communication, and feeling included in decisions affecting their work. This finding is supported by prior mixed‐methods and healthcare workplace research showing that recognition and gratitude‐based workplace cultures can improve job satisfaction, engagement, and retention [27, 29]. The theme of professional growth and compensation also emerged strongly, especially among technologists in early and mid‐career stages. This supports existing evidence that fair pay, promotion structures, and clear career development pathways are central to sustaining professional motivation in healthcare settings [4, 7, 8, 10, 12, 18]. Finally, the theme of workload and autonomy confirms the importance of balancing task demands with professional control and support. Recent radiography and workforce literature suggests that autonomy, role development, leadership, and future career pathways are increasingly important for workforce sustainability and long‐term retention [19, 20, 31–34].

Taken together, the present study contributes to the contemporary debate on job satisfaction by demonstrating that, in the Kuwaiti radiography context, job satisfaction is best understood as a multidimensional experience shaped by organizational support, compensation, leadership, communication, workload, recognition, autonomy, and professional development. This interpretation is consistent with current international evidence and highlights the need for context‐sensitive interventions that go beyond financial incentives alone.

### 4.1. Recommendations

To improve job satisfaction among DRTs in Kuwait, several targeted actions are recommended. First, implementing structured recognition and reward programs can enhance motivation, especially for non‐Kuwaiti staff who reported increased satisfaction when acknowledged. Enhancing organizational communication through regular, transparent updates and fostering open dialogue between DRTs and management can strengthen support systems. Establishing career ladder programs that provide clear promotion and development pathways is essential, particularly for technologists with 2–5 years of experience who demonstrated higher satisfaction levels. Reviewing and adjusting compensation and fringe benefits is also crucial to ensure fairness, especially for M.Sc. and Ph.D. holders who expressed dissatisfaction with their current remuneration.

Additionally, balancing workload and improving autonomy through task evaluation and increased decision‐making opportunities may address dissatisfaction among those with 6–10 years of experience. Aligning job roles with educational qualifications is necessary to meet the expectations of higher‐educated technologists and prevent the underutilization of their skills. Addressing the satisfaction gap between male and female DRTs through targeted mentorship and equal career advancement opportunities can help promote gender equity. Finally, introducing emotional and psychological support programs would help manage burnout and job‐related stress, as highlighted in both qualitative and quantitative findings. Collectively, these measures can contribute to a more satisfied and productive DRT workforce, ultimately enhancing the quality of healthcare services in Kuwait.

### 4.2. Limitations

The main limitation of this study might be including the DRTs in public hospitals and excluding the other sectors such as the military, private, oil, and petroleum, in Kuwait. In addition, DRTs in academic or medical imaging vendors were not evaluated as well. Moreover, the possible effect of nonresponse bias in the check results also must be considered.

## 5. Conclusion

Overall, the DRTs in Kuwait expressed moderate to high satisfaction levels compared to their international peers. The findings on job satisfaction urge government policymakers to concentrate their efforts on enhancing DRTs’ promotion programs and career development.

The main limitation of this study might be including the DRTs in public hospitals and excluding the other sectors such as the military, private, oil, and petroleum, in Kuwait. In addition, DRTs in academic or medical imaging vendors were not evaluated as well. Moreover, the possible effect of nonresponse bias in the check results also must be considered.

Future works, a qualitative research approach could highlight further profound comprehension and passion toward job satisfaction. Assessing leadership styles at departmental and institutional scales can shed light on their correlation with job satisfaction. The authors recommend that service directors and the general directorate of radiology at MOH conduct regular assessments on the radiography pool and use the results as a standard for ongoing comparisons. This will be essential for covering progress and detecting faults.

**Nomenclature**
DRT(s): Diagnostic Radiographic Technologist(s)
MOH: Ministry of Health
B.Sc: Bachelor of Science
M.Sc: Master of Science
Ph.D: Doctor of Philosophy
JSS: Job Satisfaction Survey
JSS‐2: Job Satisfaction Survey‐2
SPSS: Statistical Package for the Social Sciences
PACS: Picture Archiving and Communication System
USD: United States Dollar
HSC: Health Sciences Centre

## Author Contributions

Ebtehal M. Al‐Qattan and Hamad Al‐Hamad conceptualized and designed the study. Asseel Khalaf and Raed Saeed contributed to data collection and analysis. Nabeel Akhtar performed data interpretation and assisted with statistical validation. Ebtehal M. Al‐Qattan and Asseel Khalaf drafted the main manuscript text. Hamad Al‐Hamad and Nabeel Akhtar reviewed and substantively revised the manuscript.

## Funding

The authors thank the participating radiographer technologists and Kuwait University for their support of this research under Grant No. NR01/23.

## Disclosure

The views and opinions expressed in this article are those of the authors and do not necessarily reflect the official policy or position of any affiliated institution or funding body. All authors read and approved the final version of the manuscript and agreed to be personally accountable for their contributions.

## Ethics Statement

The authors confirm that the study was conducted according to the Helsinki Declaration as revised in 2013. Ethical approval for this study was obtained from the Ministry of Health in Kuwait (Approval No. 3554 from the Standing Committee for Coordination of Medical and Health Research) and from the Health Sciences Centre (HSC) Ethical Committee at Kuwait University (Approval No. VDR/EC‐369). To ensure informed consent, all participants were required to provide electronic consent before completing the questionnaire. The survey included an introductory section outlining the study’s purpose, participants’ rights, and assurances of confidentiality. No personally identifiable information was collected. Rigorous data handling and labeling procedures were followed to protect the anonymity and security of the collected information.

For the qualitative component, interviews were conducted in private, quiet settings to ensure confidentiality and minimize distractions. Each interview lasted approximately 45–60 min and was conducted by trained interviewers fluent in both Arabic and English. Participants were informed that they could skip any question or withdraw from the interview at any time without any consequences.

## Consent

Consent for publication was obtained, and the accreditation number for this research is NR01/23.

## Conflicts of Interest

The authors declare no conflicts of interest.

## Data Availability

The data that support the findings of this study are available upon request from the corresponding author. The data are not publicly available due to privacy and ethical restrictions.
